# Evaluating the Use of Seclusion Across Patient Demographics in a UK High Secure Hospital

**DOI:** 10.1192/bjo.2026.11644

**Published:** 2026-06-30

**Authors:** Laura Gröger, Sophie Gurr, Lauren Boniface, Chloe Moore, Susanna Martin

**Affiliations:** 1 Broadmoor Hospital, West London NHS Trust, Berkshire, United Kingdom; 2 South London and Maudsley NHS Foundation Trust, London, United Kingdom; 3 Kingston University, Greater London, United Kingdom

## Abstract

**Aims:**

There are growing concerns of disparities in mental health care, with suggestions of discrepancies in the use of seclusion across patients from different backgrounds. The aim of this project was to evaluate the use of short-term seclusion (STS) and long-term segregation (LTS) across patient ethnicity alongside other demographics in Broadmoor High Secure Hospital (HSH).

**Methods:**

This quantitative study analysed STS and LTS episodes from July 2022 to July 2025 alongside various demographics. STS consisted of 183 patients and 1,614 seclusions, LTS of 128 patients and 220 segregations.

**Results:**

STS:

Correlational analysis found significant relationships for admission length in duration and frequency, with longer stay patients experiencing more episodes and higher durations. Correlational analysis also identified significant relationships for patient age in duration and frequency, with older patients experiencing more episodes and higher durations. BMI significantly correlated with frequency of episodes, with higher BMI experiencing more episodes.

Inferential analysis found significant differences in frequency and duration based on where a patient was transferred from, with patients from prison experiencing higher frequency and duration than patients from medium secure units (MSU).

Significant differences were found based on index offence, with terrorist offenders experiencing higher frequencies and durations relative to patients with multiple offences or an offence of violence against the person. Patients with an alleged offence had higher durations of STS than those with multiple offences or violence against the person.

No significant differences were found between patient ethnic groups, diagnosis or religion.

LTS:

Correlational analysis identified a significant positive relationship between duration and admission length, with longer stay patients having longer durations. Additionally, a significant positive correlation was found between duration and BMI, with higher BMI relating to higher duration.

Inferential analysis found significant differences in duration between ethnic groups, with the Asian group experiencing lower durations than the Black, Mixed and White groups. A significant difference was found in duration based on where patients were transferred from, with those from prison experiencing higher durations than patients from MSU or HSH.

No significant differences were found between diagnosis, religion, index offence or age.

**Conclusion:**

The implementation of STS significantly differed based on admission length, age, BMI, where a patient was transferred from and index offence. LTS differed significantly based on patient ethnic group, admission length, where a patient was transferred from and BMI. An ongoing qualitative study aims to further elucidate these differences through staff and patient perspectives to enable clinical and research recommendations.

